# Differential Cytotoxic Function of Resident and Non-resident CD8+ T Cells in the Human Female Reproductive Tract Before and After Menopause

**DOI:** 10.3389/fimmu.2020.01096

**Published:** 2020-06-04

**Authors:** Marta Rodriguez-Garcia, Zheng Shen, Jared M. Fortier, Charles R. Wira

**Affiliations:** ^1^Department of Microbiology and Immunology, Geisel School of Medicine at Dartmouth, Lebanon, NH, United States; ^2^Department of Immunology, Tufts University School of Medicine, Boston, MA, United States

**Keywords:** menopause, female genital tract, sexually transmitted infections, tissue resident memory T cells, perforin, granzyme A, aging, TGF-β

## Abstract

The functional characterization and regulation of tissue resident and non-resident CD8+ T cells in the human female reproductive tract (FRT) as women age remains a gap in our knowledge. Here we characterized the cytotoxic activity and granular contents of CD8+ T cells from the FRT in pre- and postmenopausal women. We found that under steady-state conditions, CD8+ T cells from endometrium (EM), endocervix and ectocervix displayed direct cytotoxic activity, and that cytotoxicity increased in the EM after menopause. Cytotoxic activity was sensitive to suppression by TGFβ exclusively in the EM, and sensitivity to TGFβ was reduced after menopause. Under steady-state conditions, cytotoxic activity (measured as direct killing activity), cytotoxic potential (measured as content of cytotoxic molecules) and proliferation are enhanced in non-resident CD8+ (CD103−) T cells compared to tissue resident (CD103+) T cells. Upon activation, CD103+ T cells displayed greater degranulation compared to CD103− T cells, however the granular content of perforin, granzyme A (GZA) or granzyme B (GZB) was significantly lower. After menopause, degranulation significantly increased, and granular release switched from predominantly GZB in premenopausal to GZA in postmenopausal women. Postmenopausal changes affected both CD103+ and CD103− subpopulations. Finally, CD103+ T cells displayed reduced proliferation compared to CD103− T cells, but after proliferation, cytotoxic molecules were similar in each population. Our results highlight the complexity of regulation of cytotoxic function in the FRT before and after menopause, and are relevant to the development of protective strategies against genital infections and gynecological cancers as women age.

## Introduction

The immune system in the genital mucosa has adapted to multiple challenges unique to the human female reproductive tract (FRT), including acceptance of pregnancy, defense against sexually transmitted pathogens and tumor control. To optimize reproductive success, the immune system in the FRT is compartmentalized and regulated in a site-dependent manner by sex hormones (1). However, as women age, sex hormones decline, and women transition into menopause. In the United States, 52 is the average age for menopause, which gives women a uniquely long survival potential after their reproductive years (2).

T-cell immunity is a key arm of mucosal defense that is tightly regulated by sex hormones in the FRT (1). After menopause, when reproductive function is lost, T cell populations in the endometrium undergo changes in phenotype, distribution and function. We have previously described multiple changes in different T cell populations after menopause in the endometrium, including decreased CD4^+^/ CD8^+^ T cell ratios (3), increased Th17 CD4^+^ T cell numbers (3), decreased PD-L1 expression (4) and increased cytotoxic activity in lymphocytes (5).

In addition, we recently reported that after menopause tissue resident memory T cell (TRM) distribution changes in the endometrium and in the cervix (6). In the endometrium (EM), TRM presence increased after menopause and remained stable with age. In contrast, TRM presence progressively decreased in an age-dependent manner in endocervix (CX) and ectocervix (ECX) after menopause. TRMs are a subset of memory T cells that reside in tissues for long periods of time (7, 8). TRMs are phenotypically characterized by the expression of CD69 and, particularly on CD8+ TRMs, CD103 which allows interactions with epithelial cells (9). Long lasting residency in tissues coupled with their presence at mucosal sites, enables TRMs to provide rapid protection in response to secondary infections (10, 11). Human studies have revealed the presence of TRMs in different parts of the FRT, from vagina to endometrium (6, 12–15). However, while the mechanisms of action of TRMs in mouse models have been intensively studied, the functional characterization of TRMs in humans, particularly in the different compartments of the FRT, remains largely unknown.

Earlier studies from our laboratory with mixed cell suspensions from the FRT to measure cytotoxicity using an indirect-lysis assay, demonstrated that T cell cytotoxic activity was regulated by the menstrual cycle (5). Cytotoxic activity was lowest during the secretory phase of the menstrual cycle and increased during the proliferative phase, consistent with the need to dampen cytotoxic responses at a time when implantation by a semi-allogeneic embryo may occur (1). These earlier studies also demonstrated that cytotoxic activity was increased after menopause (5). However, detailed characterization of CD8+ T cell function in the FRT, including regulation, content of cytolytic molecules and tissue residency was not considered in these early studies. Recognizing that cytotoxic activity in the human FRT can be different from blood responses (16), detailed characterization of CD8+ T cell function in the FRT is essential to develop preventive and therapeutic strategies against genital infections and gynecological cancers.

In this study, we developed an assay to directly measure cytotoxic function of purified FRT CD8+ T cells. The goals of this study were to quantify cytotoxic activity and granular content of resident and non-resident CD8+ T cells at different sites of the FRT and evaluate the impact of menopause on CD8+ T cell function.

## Materials and Methods

### Study Subjects

Studies were performed with Dartmouth College Institutional Review Board approval. Approval to use discarded tissues from hysterectomies was obtained from the Committee for the Protection of Human Subjects (CPHS). Indications for surgery were benign conditions and tissue samples distal from the sites of pathology and without pathological lesions were selected as determined by a pathologist. Women were not on oral contraceptives before hysterectomy. Menopausal status was determined by a pathologist based on the histological evaluation of sections of the endometrium (endometrial dating). Post-menopausal status was characterized by an atrophic endometrium. Information regarding genital infections was not available.

### Tissue Processing

Tissues obtained from hysterectomies included endometrium (EM), endocervix (CX) and ectocervix (ECX) and were transferred to the laboratory immediately after surgery and processed as previously described (3, 6, 15, 17, 18). Vaginal tissues were not available. Tissues were rinsed with HBSS and minced under sterile conditions into 1–2 mm fragments and digested using an enzyme mixture containing 0.05% collagenase type IV (Sigma-Aldrich, St. Louis, MO) and 0.01% DNAse (Worthington Biochemical, Lakewood, NJ) for 1h at 37°C. Type IV collagenase was selected based on preliminary studies to ensure non-cleavage of surface markers (3, 15). After digestion, cells were dispersed through a 250 μm mesh screen (Small Parts, Miami Lakes, FL) and filtered through a 20 μm mesh screen (Small Parts) to separate epithelial cells from stromal cells. Stromal cells were then washed and counted and dead cells removed using the Dead cell removal kit (Miltenyi Biotec, Auburn, CA) according to manufacturer instructions to obtain a mixed cell suspension for flow cytometric analysis, degranulation assays and further cell purification.

### CD8^+^ T Cell Isolation

Following removal of dead cells, CD8^+^ T cells were isolated using negative magnetic bead selection with the CD8^+^ T cell isolation kit (Miltenyi Biotec) following instructions with minor modifications. This negative selection protocol delivers untouched CD3^+^CD8^+^ T cells. Additionally, anti-fibroblast microbeads (Miltenyi Biotec) were added in combination with the microbeads supplied with the kit to ensure depletion of stromal fibroblasts present in the mixed cell suspension as described before for CD4 selection (3). After two rounds of negative selection, purity of the CD8^+^ T cell population was higher than 90%, with ~2% contamination with non-immune cells, 2% CD3- cells and 1–2% contamination with CD4+ T cells (Supplementary Figure 1A). Following isolation, CD8+ T cells were used in cytotoxicity assays without *in vitro* stimulation, or stimulated for degranulation and proliferation assays.

### CD103− and CD103+ CD8+ T Cell Isolation

Purified CD8+ T cells were incubated with CD103−PE antibody (Miltenyi) for 10 min, followed by incubation with anti-PE ultra-pure beads (Miltenyi) to separate CD103+ cells by positive magnetic separation, and CD103− by negative selection.

### Cytotoxicity Assay

Purified CD8+ T cells (or CD103+ or CD103− as indicated) were co-cultured with CFSE-stained (Cell Division Tracker Kit; BioLegend) allogeneic blood CD4+ T cells, at a Effector:Target ratio of 1:1, in 96-well plates. Cytotox red (IncuCyte Cytotox Red, Essen Bioscience) was added to the media to stain dead cells. Plates were imaged every 10 min using the IncuCyte Zoom system (Essen Bioscience), and dead target cells were automatically quantified over time as double green (CFSE) and red (Cytotox) stained cells. For some experiments, purified CD8+ T cells were pre-treated for 2 h with TGFβ (10 ng/ml, PeproTech Inc) or TGFβ Receptor 1 blocker, SB431542 (10 μM, Tocris Cookson Inc) (19) prior to co-culture with target cells.

### Degranulation Assay

Mixed cell suspensions were activated with phorbol 12-myristate 13-acetate (PMA) (100 ng/ml, Abcam) and ionomycin (2 μM, Calbiochem) for 1 h in the presence of CD107a-PE-Cy7 (BD Bioscience) antibody, followed by 4 additional hours in the presence of Brefeldin A (BD GolgiPlug protein transport inhibitor, BD Biosciences) as described before (20), surface stained and fixed and permeabilized with the BD Cytofix/Cytoperm kit (BD Biosciences) according to the instructions. Intracellular staining of perforin, GZA and GZB was performed as described below.

### Flow Cytometry

Mixed cell suspensions were stained for surface markers with combinations of the following antibodies: CD45-vioblue 450, CD8-FITC (Tonbo), CD3-viogreen (Miltenyi), CD45-AF700, CD3-APC-Cy7, CD4-APC-Cy7, CD103–BV711 (Biolegend), CD4-PE-Cy5.5, CD103–PE-Cy7 (eBioscience, San Diego, CA), CD8-BUV395 (BD Bioscience). Analysis was performed on BioRad ZE5 flow cytometers (BioRad) using Everest software or Gallios (Beckman Coulter) using Kaluza software, and data analyzed with FlowJo software (Tree Star, Inc. Ashland, OR). Expression of surface markers was measured by the percentage of positive cells.

### Intracellular Staining

Detection of perforin, GZA and GZB was performed on mixed cell populations after dead cell removal or after stimulation of cells in the degranulation assay. Cells were surface stained first and then fixed and permeabilized with Cytofix/cytoperm kit (BD) according to instructions. Intracellular staining of perforin, Granzyme A and B were done using combinations of the following antibodies: anti-human Perforin-PE/Dazzle, Granzyme A-AF647, Granzyme A-PerCp-Cy5.5, Granzyme B-AF647 (Biolegend) and Granzyme B-BV421 (BD Bioscience).

### Proliferation Assay

Purified CD103+ and CD103− CD8+ T cells were stained with CFSE and stimulated with anti CD3/CD28 beads (Dynabeads Human T-Activator CD3/CD28, Gibco) as recommended by the manufacturer to induce proliferation. Cells were incubated in 96-well round-bottom plates for 4 days and evaluated by flow cytometry after intracellular staining as described above.

### Statistics

Data analysis was performed using the GraphPad Prism 5.0 software. A two-sided *P*-value < 0.05 was considered statistically significant. Comparison of two groups was performed with the non-parametric test U–Mann Whitney or Wilcoxon paired test. Comparison of three or more groups was performed applying the non-parametric Kruskal-Wallis test followed by Dunns post-test.

## Results

### Characterization of Direct Cytotoxic Activity in CD8+ T Cells From the FRT

**Figure 1:**
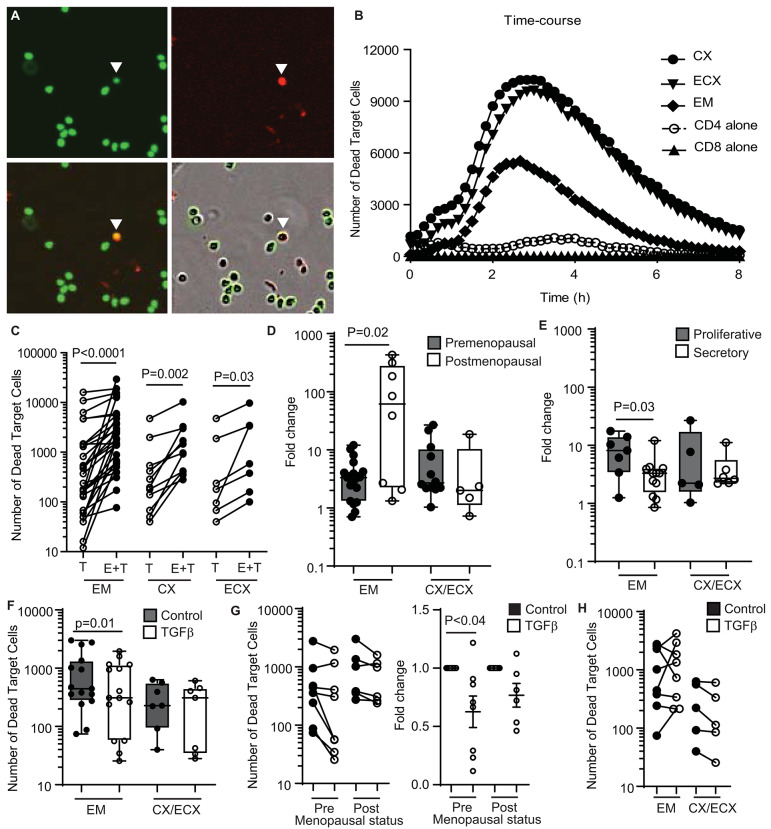
Quantification and regulation of cytotoxic activity in CD8+ T cells from the FRT in premenopausal and postmenopausal women**. (A)** Representative image showing each fluorescent channel and the merge of CFSE-stained CD4+ T cells (upper left: green; target cells), dead cells (upper right: red; cytotox) and dead target cells (lower left: yellow; CFSE + cytotox). A dead target cell is identified in the images with a white arrow. Bottom right image shows phase contrast and fluorescent signals. **(B)** Representative time-course of the kinetics of cytotoxicity over a period of 8 h using a E:T ratio of 1:1. Each curve represents the number of dead target cells using purified CD8+ T cells from the endometrium (EM), endocervix (CX) and ectocervix (ECX) from the same patient as indicated. CD4 alone are allogeneic CD4+ T cells isolated from blood. CD8 alone are purified CD8+ T cells from EM. **(C)** Mean number of dead target cells in the target alone condition (T) compared to effector + target cells (E+T) in 4 h. Each dot represents a different patient (*N* = 31 for EM; *N* = 14 for CX; *N* = 7 for ECX). **(D)** Comparison of pre vs. postmenopausal women. Graph represents the fold change in number of dead target cells in E+T condition compared to target cells alone. Effector CD8+ T cells from endocervix and ectocervix are shown combined. **(E)** Comparison of endometrial CD8+ T cells from premenopausal women in the proliferative or secretory phases of the menstrual cycle. **(F)** Mean number of dead target cells during 4 h in the absence (control) or presence of TGFβ in the endometrium (EM = 15) or cervix (CX/ECX = 7). **(G)** Comparison of the effect of TGFβ treatment in premenopausal (*n* = 9) and postmenopausal (*n* = 6) women in the total number of dead target cells (left graph) and fold change in the mean number of dead target cells after TGFβ treatment compared to control (right graph). **(H)** Number of dead target cells after TGFβ-signaling blockade treatment compared to control in endometrium (EM = 8), endocervix and ectocervix (CX/ECX = 5).

We previously demonstrated the presence of cytotoxic activity in mixed cell suspensions from the FRT using a redirected killing assay (5). Here we wanted to evaluate the direct cytotoxic activity of purified CD8+ T cells throughout the FRT. We developed an image-based *in vitro* assay to directly measure cytotoxic activity of purified CD8+ T cells from EM, CX and ECX. CD8+T cells were co-cultured with allogeneic CFSE-stained blood CD4+T cell targets and cytotoxicity was measured over time using quantitative time-lapse microscopy. As shown in Figure 1A (upper left), CD4+ target T cells can be identified by green staining (CFSE). To detect dead cells, a cell-impermeant DNA dye was added to the culture media, which labels cells in red when the membrane is disrupted (upper right). When CFSE-labeled CD4+ T cells are killed (lower left), their color changes from green to yellow, as a result of the overlay of green and red signals. Yellow cells can be quantified to determine the number of dead target cells. Bottom right image is a composite showing both phase contrast and fluorescent signals.

Using this approach, we observed a dose-response effect in cell killing when increasing numbers of CD8+ T cells were added (Supplementary Figure 1B). Based on this, we selected an Effector:Target ratio of 1:1 for further experiments. We also determined the kinetics of killing, which started shortly after initiation of the assay, and reached a peak between 2 and 4 h (Figure 1B). We therefore chose to quantify the average cell killing within the first 4 h, which offered a clear killing signal above background cell death noise (Figure 1B). Using these parameters, we detected a significant increase, relative to target cells alone, in the number of dead target cells in the presence of allogeneic CD8+ T cells from the EM, CX, and ECX (Figure 1C).

### Comparison of Direct Cytotoxic Activity of CD8+ T Cells From the Upper and Lower FRT of Pre- and Postmenopausal Women

Previously, using a redirected-cell lysis assay with mixed cell suspensions, we reported a cyclic pattern of endometrial cytotoxicity that changed with stage of menstrual cycle and menopause; no evidence of cyclicity was found with CD8+ T cells from the endocervix or ectocervix (5). Therefore, here we investigated whether menstrual and menopausal status directly regulate CD8+ T cell cytotoxic activity. For this analysis, we normalized the data shown in Figure 1C to express cytotoxicity as the fold change in number of dead target cells, to be able to compare between experiments with different background mortality in the target cell alone control. Following normalization, we stratified the patients according to pre- and postmenopausal status (Figure 1D). Postmenopausal women displayed greater endometrial CD8+ T cell cytotoxicity when compared to premenopausal women. In contrast, menopausal status had no effect on cervical and ectocervical CD8+ T cell killing (Figure 1D). Next, we divided premenopausal women into proliferative or secretory according to their stage of the menstrual cycle (Figure 1E). Confirming our earlier results (5), CD8+ T cell cytotoxic activity was significantly reduced in the EM during the secretory phase compared to the proliferative phase of the cycle, with no significant changes detected in CX and ECX.

### CD8+ T Cell Cytotoxic Activity Is Regulated by TFGβ in EM but Not in CX and ECX

TGFβ is synthesized and secreted by epithelial cells and stromal cells in the FRT (21, 22) and has been previously described in mice to suppress cytotoxic activity of CD8+ T cells *in vitro* (23). Therefore, we investigated whether TGFβ might be implicated in regulating FRT cytotoxic activity.

Purified FRT CD8+ T cells were pre-treated with TGFβ for 2h before co-culture with CFSE-stained allogeneic CD4+ T cells, with cell killing evaluated as detailed above (Supplementary Figure 1C). As shown in Figure 1F, TGFβ pretreatment reduced the killing capacity of endometrial CD8+ T cells; however, TGFβ treatment had no effect on CD8+ T cells from CX and ECX. Since we had observed differences in cytotoxic activity between pre- and postmenopausal women (Figure 1D), we evaluated whether TGFβ suppression was dependent on menopausal status. We stratified the subjects according to their menopausal status and, as shown in Figure 1G (left graph), the same suppression trend after TGFβ treatment was observed in pre- and postmenopausal women. To control for variation between individuals in the pre- and postmenopausal groups, we then calculated the fold change in cell killing after TGFβ treatment. As shown in Figure 1G (right graph) TGFβ pretreatment significantly suppressed cytotoxicity in CD8+ T cells from premenopausal women, but postmenopausal women displayed a non-significant trend toward suppression, suggesting a decreased sensitivity to the effects of TGFβ.

Since we observed that TGFβ decreased killing by CD8+ T cells from the endometrium, we next explored whether blocking TGFβ signaling would increase cytotoxic activity. Purified CD8+ T cells were pre-treated with SB431542 (a selective inhibitor of TGFβ receptor signaling) for 2 h prior to coculture with CFSE-labeled allogeneic target cells. TGFβ-signaling blockade was able to increase the cytotoxic activity of endometrial CD8+ T cells in some individuals (Supplementary Figure 1D); however, this effect was not consistent, with only a subset of patients being sensitive to blockade (Figure 1H). In contrast to the EM, no effect was detected for CD8+ T cells from CX and ECX (Figure 1H).

### CD103+ CD8+ T Cells Have Reduced Direct Cytotoxic Activity Compared to CD103− CD8+ T Cells

**Figure 2:**
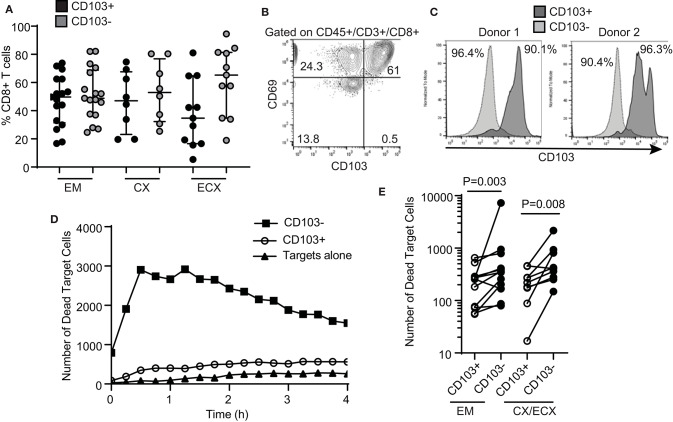
Comparison of cytotoxic activity between CD103^+^ and CD103^−^ CD8^+^ T cells. **(A)** Percentage of CD103^+^ and CD103^−^ cells in the CD8+ T cell population in the endometrium (EM), endocervix (CX) and ectocervix (ECX). **(B)** Expression of CD103 and CD69 on CD8+ T cells from the FRT. Representative example from 4 different samples. **(C)** Purity of CD103^+^CD8^+^ (dark gray) and CD103^−^CD8^+^ (light gray) T cell populations after magnetic bead selection. Each histogram overlay shows the purity of both populations from the same patient. Two representative examples are shown. Cells were gated on CD45+, CD3+ and CD8+. **(D)** Representative example of dead target cell kinetics with purified CD103^−^CD8^+^ (squares) and CD103^+^CD8^+^ (white circles) T cells from the same patient. **(E)** Comparison of mean number of dead target cells in the presence of purified CD103+ or CD103− CD8+ T cells. Each dot represents a different subject.

We and others have previously demonstrated that human FRT CD8+ T cells can be classified into tissue resident cells and non-resident CD8+ T cells (6, 12, 13). Tissue resident CD8+ T cells are phenotypically characterized by the expression of CD69, with or without co-expression of CD103 (8). In the present study, we observed a distribution of CD103+ and CD103− CD8+ T cells consistent with our previous report (6), with a median percentage of CD103+ CD8+ T cells of 50, 47, and 35% of the total CD8+ T cells for EM, CX and ECX, respectively (Figure 2A). Further, we confirmed that in the FRT, almost all CD103+ CD8+ T cells co-express CD69, while CD103− CD8+ T cells represent a mixture of CD69+ and CD69− cells (Figure 2B).

Tissue resident memory T cells have been implicated in protection against genital infections such as HSV-2 and HPV in animal models (24–26). Therefore, we asked whether human tissue resident CD103+ CD8+ T cells and CD103− CD8+ T cells from the FRT display differential cytotoxic activity. As described in Methods, we optimized a protocol to isolate CD103+ and CD103− CD8+ T cells from the FRT using sequential magnetic bead selection, and obtained two purified CD8+ T cell populations from the same patients (CD103+ and CD103−), with a purity range between 90 and 96% (Figure 2C).

Using the cytotoxicity assay described above for total CD8+ T cells, we compared the ability of purified CD103+ and CD103− CD8+ T cells from the same patient to kill allogeneic target cells. Our initial hypothesis was that CD103+ T cells would have greater killing capacity when compared to matched CD103− CD8+ T cells. Unexpectedly, we found that cytotoxic activity was significantly lower in CD103+ CD8+ T cells when compared to CD103− CD8+ T cells (Figure 2D, curve comparison). We found this difference throughout the FRT, with CD103− CD8+ T cell cytotoxic activity greater than that seen with matched CD103+ CD8+ T cells from the EM, CX and ECX (Figure 2E, all patients).

### Reduced Cytotoxic Potential in CD103+ Compared to CD103− CD8+ T Cells

**Figure 3:**
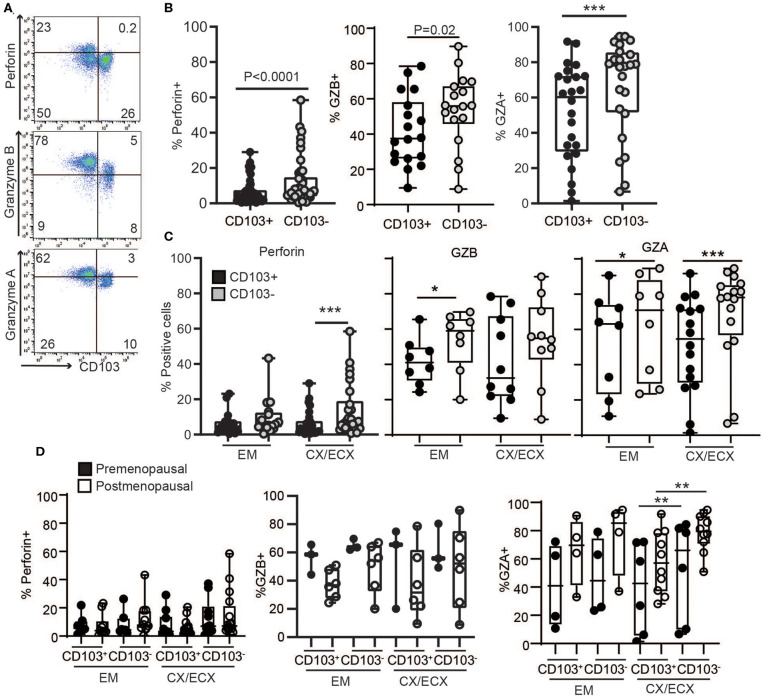
Comparison of intracellular cytotoxic molecules in resting CD103^+^ and CD103^−^ CD8^+^ T cells. **(A)** Representative dot plots and **(B)** graphs of intracellular levels of perforin, granzyme B (GZB) and granzyme A (GZA) in CD103+ and CD103− CD8+ T cells. **(C)** Comparison of intracellular levels of perforin, granzyme B (GZB) and granzyme A (GZA) in CD103+ and CD103− CD8+ T cells in the endometrium (EM), endocervix and ectocervix (CX/ECX). **(D)** Comparison of pre- vs. postmenopausal (white) women. Each dot represents a different patient. *p < 0.05, **p < 0.01, ***p < 0.001.

To understand the mechanisms behind the unexpected difference in cytotoxic activity between CD103+ and CD103− CD8+ T cells, we first evaluated the content, under resting conditions, of cytotoxic molecules in freshly isolated CD103+ and CD103− CD8+ T cells from the FRT. EM, CX, and ECX tissues were digested to obtain mixed cell suspensions and stained for the intracellular cytotoxic molecules perforin, granzyme B (GZB) and granzyme A (GZA) for analysis by flow cytometry as detailed in methods. As seen in Figures 3A,B, we found that, compared to CD103− CD8+ T cells, CD103+ cells had significantly reduced levels of perforin, GZB and GZA under resting conditions. To explore whether these differences were site-dependent, we next compared CD8+ T cells from endometrium and cervix. As shown in Figure 3C, while GZA expression was greater in CD103− than in CD103+ CD8+ T cells in all FRT compartments, we observed site-dependent differences for perforin and GZB, significantly increased in CD103− CD8+ T cells from CX/ECX and EM, respectively.

We next investigated potential changes in cytotoxic molecules due to menopausal status (Figure 3D). Overall, no significant differences were detected between pre- and postmenopausal women within the CD103+ and CD103− CD8+ T cell compartments. While we observed a trend toward decreased expression of GZB and increased expression of GZA after menopause in both CD103+ and CD103− CD8+ T cells, these differences were not statistically significant. Although these findings suggest that levels of cytotoxic molecules in resting CD8+ T cells are not affected by menopausal status, given the limited number of samples and high variability for some groups, these results need to be confirmed in larger cohorts.

### Increased Granule Release of Cytotoxic Molecules Following Stimulation of Non-resident CD8+ T Cells

**Figure 4:**
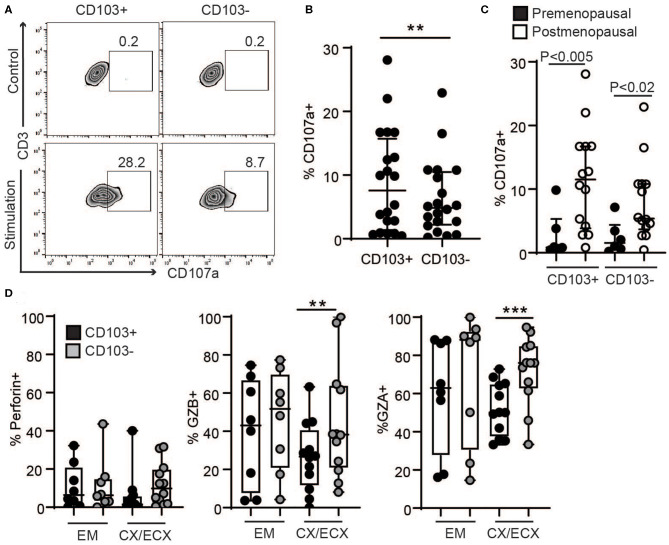
Comparison of degranulation and release of cytotoxic molecules in CD103+ and CD103− CD8+ T cells after activation. **(A)** Representative dot plot and **(B)** graph of CD107a expression in CD103+ and CD103− CD8+ T cells after activation. EM, CX, and ECX are shown combined. **(C)** Comparison of pre and postmenopausal women for CD107a expression. **(D)** Comparison of the percent of cells expressing perforin, granzyme B (GZB) and granzyme A (GZA) in CD103+ and CD103− CD8+ T cells from the endometrium (EM), endocervix and ectocervix (CX/ECX). Each dot represents a different patient. **p < 0.01, ***p < 0.001.

Since tissue resident T cells (CD103+ T cells) have been reported to be important for protection against re-infection (10), we next compared the ability of CD103+ and CD103− CD8+ T cells to release their granules in response to activation in a degranulation assay. To quantify degranulation, mixed-cell suspensions were activated with CD3/CD28 beads in the presence of CD107a antibody (LAMP-1), a major constituent of lysosomal membranes and a marker for degranulation (20). As seen in Figure 4A, after activation, a subset of CD103+ and CD103− CD8+ T cells expressed CD107a. However, CD107a was expressed in a higher percentage of CD103+ compared to CD103− CD8+ T cells (Figures 4A,B), indicating greater degranulation in CD103+CD8+ T cells. Furthermore, degranulation was significantly increased after menopause in both CD103+ and CD103− CD8+ T cells (Figure 4C).

To evaluate cytotoxic granular content, following activation for 1 h, granule release was blocked with brefeldin for 4 additional hours as described previously (20), and the presence of the cytotoxic molecules perforin, GZB and GZA was analyzed in the different anatomical sites. We found a greater percentage of CD103− T cells expressing GZB and GZA in the CX and ECX compared to CD103+ CD8+ T cells (Figure 4D), with a similar trend but no statistical difference for perforin (Figure 4D). In contrast, no differences were detected between CD103+ and CD103− cells from EM (Figure 4D).

### Distinct Profiles of Granzyme Release Before and After Menopause

**Figure 5:**
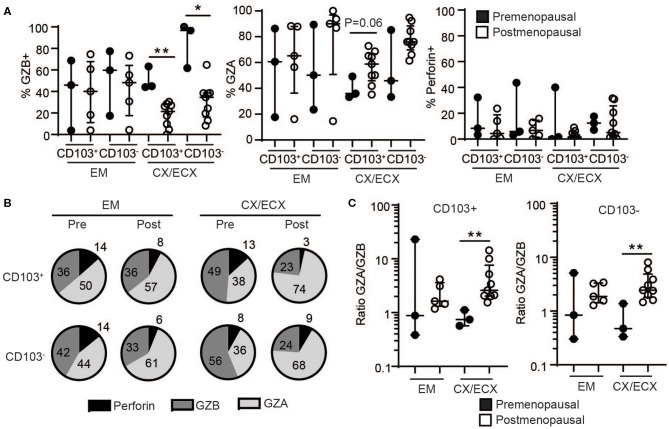
Effect of menopausal status on release of cytotoxic molecules in CD103+ and CD103− CD8+ T cells after activation. **(A)** Comparison of the percent of cells expressing granzyme B (GZB), granzyme A (GZA), and perforin in CD103+ and CD103− CD8+ T cells from the endometrium (EM), endocervix and ectocervix (CX/ECX) in premenopausal and postmenopausal women. Each dot represents a different patient. **(B)** Pie chart representing the mean value of the percentage for all the patients shown in **(A)**. **(C)** Changes in the ratio of granzyme A: granzyme B (GZA/GZB) in CD103+ and CD103− CD8+ T cells from the endometrium (EM), endocervix and ectocervix (CX/ECX) in premenopausal compared to postmenopausal women. *p < 0.05, **p < 0.01.

Next, we explored potential differences in the content of the released granules after activation of resident and non-resident CD8+ T cells between pre- and postmenopausal women. As seen in Figure 5A, CD8+ T cells from the cervix of postmenopausal women showed a significant decrease in GZB and a strong trend toward increased GZA, regardless of CD103+ or CD103− expression. In contrast, no changes related to menopausal status were detected for perforin, or for any of the molecules analyzed in the EM likely due to the high variability between samples. Specific changes in the proportions of GZA and GZB expressing cells after menopause are visualized in Figure 5B, showing a pie chart of the mean percentage expression level of perforin, GZB and GZA for all the patients in each group. Figure 5B indicates a dramatic increase in GZA (CD103+ cells: 38 to 74%; CD103− cells: 36 to 68%) and decrease in GZB (CD103+ cells: 49 to 23%; CD103− cells: 56 to 24%) exclusively in CX and ECX from postmenopausal compared to premenopausal women. Considering these observations, we then calculated the ratio between GZA and GZB expressing cells in pre- and postmenopausal women, and detected a significant increase in GZA/GZB ratio in cervical CD103+ and CD103− T cells from postmenopausal women compared to premenopausal women, with no changes in the EM (Figure 5C).

### CD103+ T Cells Display Reduced Proliferation When Compared to CD103− T Cells

**Figure 6:**
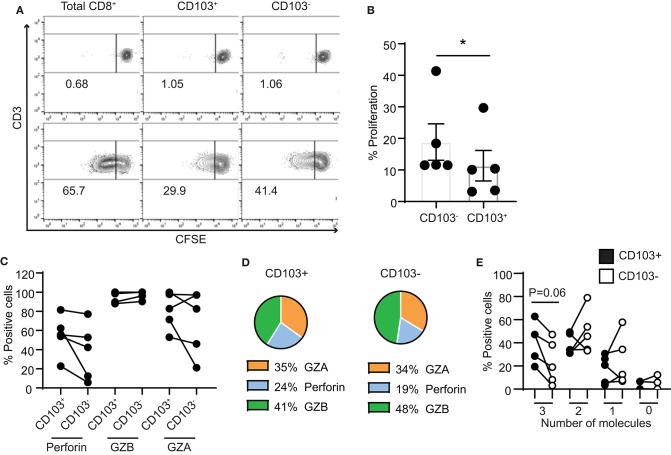
Proliferation capacity of CD103+ and CD103− CD8+ T cells. **(A)** Representative example and **(B)** graph of proliferation of purified CD103+ and CD103− CD8+ T cells from endometrium 4 days after activation with CD3/CD28 beads. **(C)** Graphs and **(D)** pie chart of the intracellular contents of perforin, granzyme A and granzyme B after 4 days of proliferation. **(E)** Comparison of CD103+ and CD103− cells after 4 days of proliferation for the percentage of cells that simultaneously produce 3 cytotoxic molecules (perforin, GZA and GZB), 2 molecules (either combination of 2 of the cytotoxic molecules), 1 molecule (only perforin, GZA or GZB) or none of the molecules analyzed (0 molecules). *p < 0.05.

Lastly, we assessed the proliferative capacity of CD103+ and CD103− CD8+ T cells in response to stimulation. We measured proliferation of purified CD103+ and CD103− CD8+ T cell populations after stimulation with CD3/CD28 beads. After 4 days, both populations proliferated in response to bead stimulation (Figure 6A), however, as seen in Figure 6B, CD103− CD8+ T cell proliferation was significantly greater than that seen with CD103+ CD8+ T cells.

As a part of this study, intracellular content of cytotoxic molecules was measured (Figure 6C). In contrast to our findings with freshly isolated cells, after 4 days of proliferation no differences were measured in the percentage of perforin, GZB and GZA positive CD103+ and CD103− cells (Figure 6D). We also analyzed the ability of CD103+ and CD103− CD8+ T cells to simultaneously produce several of the cytotoxic molecules. As shown in Figure 6E, after 4 days of proliferation, CD103+ T cells had increased ability to produce three cytotoxic molecules (perforin, GZA and GZB) when compared to CD103− T cells, although this difference did not reach statistical significance. Since the content of perforin was greater in both populations than that seen in freshly isolated cells (Figure 4), these results suggest that in response to proliferation, cytotoxic potential of CD103+ cells is increased.

## Discussion

Here we investigated the cytotoxic activity of resident and non-resident CD8+ T cells and the impact of menopausal status on cytotoxic function. We demonstrate that CD103+ T cells had reduced cytotoxic activity and reduced contents of perforin, GZA and GZB when compared to CD103− T cells. We also demonstrate site-specific differences between endometrium and cervix regarding regulation of cytotoxic activity and potential, and the impact of menopause on cytotoxic activity at each of these sites. Overall our results highlight the complexity of regulation of CD8+ T cells by the tissue environment in the FRT and menopausal status.

In previous studies, we demonstrated that TRM distribution in the FRT changes with menopause (6). The current study provides a further understanding of CD8+ T cell immune responses in the FRT by defining the impact of menopause on cytotoxic T cell function. We observed increased direct cytotoxic activity in endometrial CD8+ T cells from postmenopausal women relative to those from premenopausal women, with no effects of menopause observed in the cervix. These results confirm earlier observations from our group using a re-directed lysis assay indicating that endometrial cytotoxic activity was increased in postmenopausal compared to premenopausal women (5). In the present study we further demonstrate that endometrial cytotoxic function is susceptible to suppression by TGFβ after short-time treatment, but that sensitivity to suppression is reduced after menopause. These findings of regulation of endometrial cytotoxic function by TGFβ provide insight into our understanding of cytotoxic CD8+ T cell function in the EM in premenopausal women. We and others have demonstrated that TGFβ is produced by epithelial cells and fibroblasts from the FRT and under hormonal control (21, 22). Interestingly, under the control of sex hormones, TGFβ production is greatest during the secretory phase of the menstrual cycle, the time we find cytotoxic activity in the endometrium to be most markedly suppressed (27). Another source of TGFβ are the high levels found in semen, which is known to induce a mild inflammatory response that prepares the endometrium for implantation (28). Mechanistically, it is likely that both sources play important roles in preventing T-cell mediated allogeneic rejection of sperm.

In contrast to the EM, cytotoxic activity in the cervix was not sensitive to suppression by TGFβ. This suggests there is an underlying mechanism to ensure immune protection against sexually transmitted pathogens that enter the cervix and lower tract at a time when immune suppression is taking place in the upper tract. Our findings of TGFβ suppression of endometrial but not cervical CD8+ T cells adds to our understanding of site-specific differences in the human FRT (3, 4, 6). Others have found differential regulation of TRM cells by TGFβ at other mucosal sites. For example, unlike TRM cells in the lung, cells from the nasal mucosa develop independently of TGFβ signaling (29). Regulation of CD8+ T cell cytotoxic function by TGFβ has also been previously described for intestinal CD8+ T cells, through the suppression of perforin, without changes in T-bet, Eomes or GZB (30). Further studies are needed to identify the underlying mechanisms involved in the differential susceptibility to TGFβ between endometrium and cervix, and the decreased sensitivity to suppression by TGFβ in the EM following menopause. The potential implication of differential expression of TGFβ receptors on CD8+ T cells from the FRT, or changes in signaling pathways induced by the tissue environment or sex hormones deserve further investigation.

Recognizing the reported role of CD103+ TRMs in local protection against infections and tumor protection in animal models (24, 26, 31), we hypothesized that CD103+ TRMs would have increased direct cytotoxic activity compared to that seen with CD103− CD8+ T cells. Unexpectedly, we found that CD103− T cells had greater direct cytotoxicity than CD103+ CD8+ T cells. This finding was consistent with our demonstration that CD103− CD8+ T cells had increased levels of cytotoxic molecules (perforin, GZB and GZA) both under resting conditions and in response to degranulation when compared to CD103+ CD8+ T cells. Importantly, while CD103+ T cells in our study co-expressed CD69, likely representing intraepithelial TRMs, the CD103− population was a mixture of CD69+ and CD69− T cells, likely containing both resident (CD103−CD69+) and non-resident (CD103− CD69−) T cells. Since our selection protocol was based on CD103 selection, the potential differences in cytotoxic activity between CD103−CD69+ resident T cells and CD103−CD69− non-resident T cells could not be evaluated. Future studies are needed to address this question.

Of note, although CD103− CD8+ T cells displayed greater content of cytotoxic molecules, CD103+ TRMs expressed higher levels of CD107a+ cells after stimulation, suggesting increased ability to degranulate. Our findings suggest that the granular contents in CD103+ TRMS are not cytotoxic because of their reduced ability to kill target cells; these findings are consistent with previous reports in mouse models demonstrating a key role for TRMs in cytokine secretion and initiation of mucosal immune responses (32, 33). Similarly, human intestinal CD8+ T cells have also been shown to release granules with no cytotoxic content (34). Although our study was focused on cytotoxic activity and production of the cytotoxic molecules perforin, GZA and GZB, it is possible that CD103+ TRMs in the FRT produce IFNγ, which was not evaluated in our study, but has been described in other sites and animal models (35). Further, alternative functions of TRMs other than cytotoxicity need to be evaluated in the human FRT.

A novel finding in our study is that menopause equally affects CD103+ and CD103− T cells and has a dramatic effect on T cell degranulation. Menopause increased degranulation capacity in both resident and non-resident cells and significantly altered the composition of GZs expressed in both T cell populations, with a predominance of GZA expressing cells over GZB expressing cells. To the best of our knowledge, this is the first time that a change in GZ composition has been described as a consequence of aging. In addition to cytotoxic activity, GZs are known to have proinflammatory properties, particularly GZA, whose role in cytotoxicity is under debate (36, 37). Altered balance in GZ composition in postmenopausal women may contribute to increased inflammation in the genital tract.

Interestingly, this change in GZ composition was only observed in the cervix and did not affect overall killing activity after menopause. However, our study's ability to detect differences in cytotoxic molecules in the endometrium between pre- and postmenopausal women was limited, due to high variability between samples and low number of individuals per group. Further investigation of the consequences and mechanisms involved in GZ changes after menopause are warranted.

In conclusion, we demonstrate differences in cytotoxic function between CD103+ and CD103− CD8+ T cells as well as menopausal regulation of cytotoxic activity and potential between endometrium and cervix that is separate and distinct. Our results are relevant to understand control of cytotoxic activity in pre- and postmenopausal women and to the design of therapeutics for sexually transmitted infections and gynecological cancers.

## Data Availability Statement

The datasets generated for this study are available on request to the corresponding author.

## Ethics Statement

The studies were performed with Dartmouth College Institutional Review Board approval. Approval to use discarded tissues from hysterectomies was obtained from the Committee for the Protection of Human Subjects (CPHS).

## Author Contributions

MR-G and CW designed the research and wrote the manuscript. MR-G, ZS, and JF conducted experiments and acquired data and analyzed data. All authors approved the manuscript.

## Conflict of Interest

The authors declare that the research was conducted in the absence of any commercial or financial relationships that could be construed as a potential conflict of interest.

## Supplementary Material

Supplementary Figure 1 **(A)** Purity of CD8+ T cells after two rounds of magnetic bead selection. Low levels of contamination come from non-immune cells (CD45-), CD3- cells and CD4+ T cells and double negative T cells (DN). **(B)** Dose-response assay with increasing proportions of effector cells (E: CD8+ T cells) relative to target cells (T: CD4+ T cells). Bars represent the mean number of dead target cells during 4 h. Representative of 3 independent experiments. **(C)** Representative example of dead target cell kinetics in the presence or absence of TGFβ. **(D)** Representative example of dead target cell kinetics in the presence or absence of TGFβ-signaling blockade.
