# Supplementary figures and images for: Differential Cytotoxic Function of Resident and Non-resident CD8+ T Cells in the Human Female Reproductive Tract Before and After Menopause
Source: Front Immunol. 2020 Jun 4;11:1096. doi: 10.3389/fimmu.2020.01096 (PMC7287154; doi:10.3389/fimmu.2020.01096)

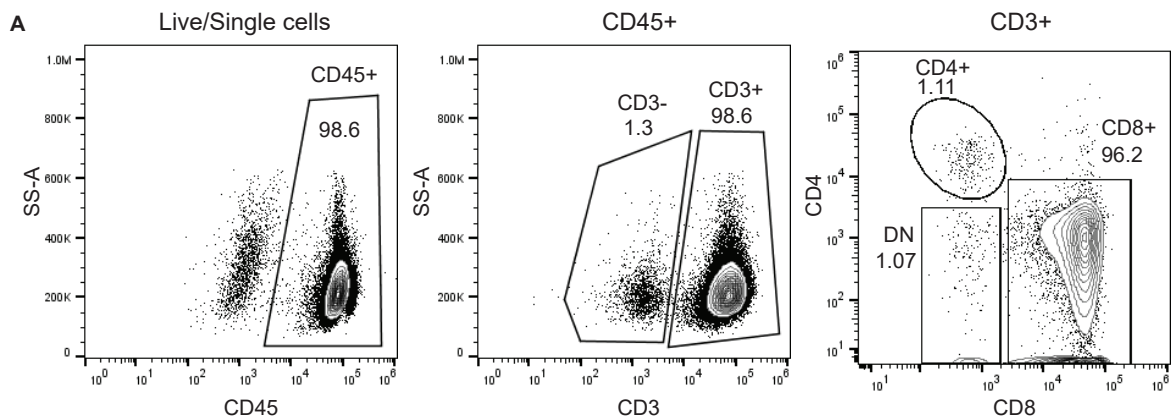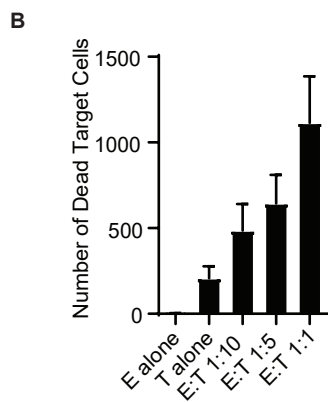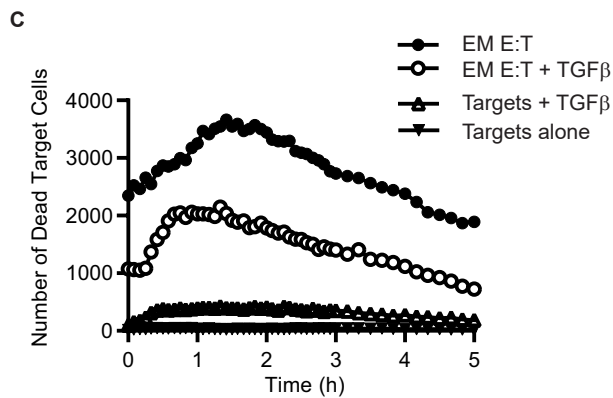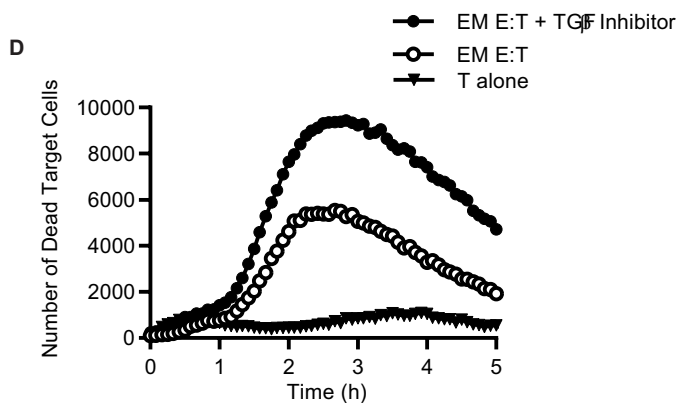

Supplement: Supplementary Figure 1 — (A) Purity of CD8+ T cells after two rounds of magnetic bead selection. Low levels of contamination come from non-immune cells (CD45-), CD3- cells and CD4+ T cells and double negative T cells (DN). (B) Dose-response assay with increasing proportions of effector cells (E: CD8+ T cells) relative to target cells (T: CD4+ T cells). Bars represent the mean number of dead target cells during 4 h. Representative of 3 independent experiments. (C) Representative example of dead target cell kinetics in the presence or absence of TGFβ. (D) Representative example of dead target cell kinetics in the presence or absence of TGFβ-signaling blockade. [file Data_Sheet_1.PDF]
